# Supplementary material for: TNF controls a speed-accuracy tradeoff in the cell death decision to restrict viral spread
Source: Nat Commun. 2021 May 20;12:2992. doi: 10.1038/s41467-021-23195-9 (PMC8137918; doi:10.1038/s41467-021-23195-9)
Supplement: Supplementary file 1 — Supplementary Information [file 41467_2021_23195_MOESM1_ESM.pdf]

Supplementary Information

TNF controls a speed-accuracy tradeoff in the cell death decision to restrict viral spread

Authors: Jennifer Oyler-Yaniv[1,=], Alon Oyler-Yaniv[1,=], Evan Maltz[1], Roy Wollman[1,2,3*]
[1]Institute for Quantitative and Computational Biosciences, University of California, Los Angeles.
[2]Department of Integrative Biology and Physiology, University of California UCLA.
[3]Department of Chemistry and Biochemistry, University of California UCLA.
These authors contributed equally: Jennifer Oyler-Yaniv, Alon Oyler-Yaniv
[*]Corresponding author: rwollman@ucla.edu

Figure S1

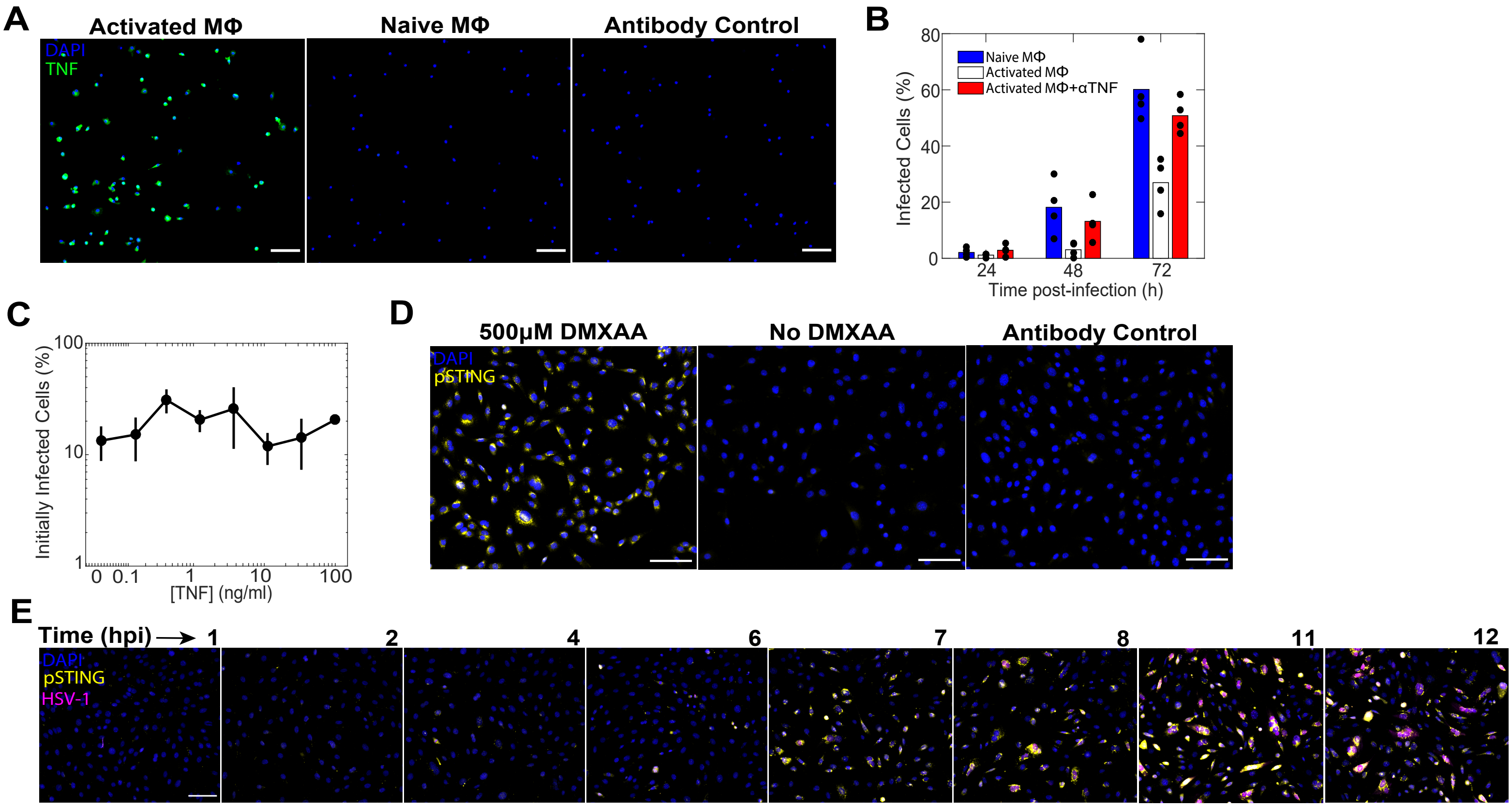

Supplementary Figure 1 (Related to Figure 1): TNF production by activated macrophages restricts viral spread, but not through mechanisms of infectivity or viral sensing. A, Representative images of mouse BMDM activated with IFN and LPS, and with Brefeldin A for 5 hours. BMDM were then fixed, permeabilized and stained with anti-TNF. B, Naive or activated BMDM were co-cultured with 3T3 fibroblasts, infected with MOI 1 HSV-1, and, where indicated, supplemented with neutralizing anti-TNF antibodies. The percentage of infected 3T3 fibroblasts were quantified at 24, 48, and 72hpi based on the intensity of tagged viral VP26. Bars denote the mean of replicates (filled black circles). C, Quantification of 3T3 cells infected only from virus in the media after being pulsed overnight with dose titrations of TNF and infected with MOI 2 of HSV-1. Viral infection was detected based on the intensity of tagged viral VP26. Data are mean ± s.d. D, Representative images of 3T3 fibroblasts stained for pSTING after being pulsed overnight with TNF, washed, and exposed to the synthetic STING agonist DMXAA. E, Representative images of 3T3 fibroblasts stained for pSTING after being pulsed overnight with TNF, washed, and infected with MOI 10 of HSV-1. All scale bars,100μm.

Figure S2

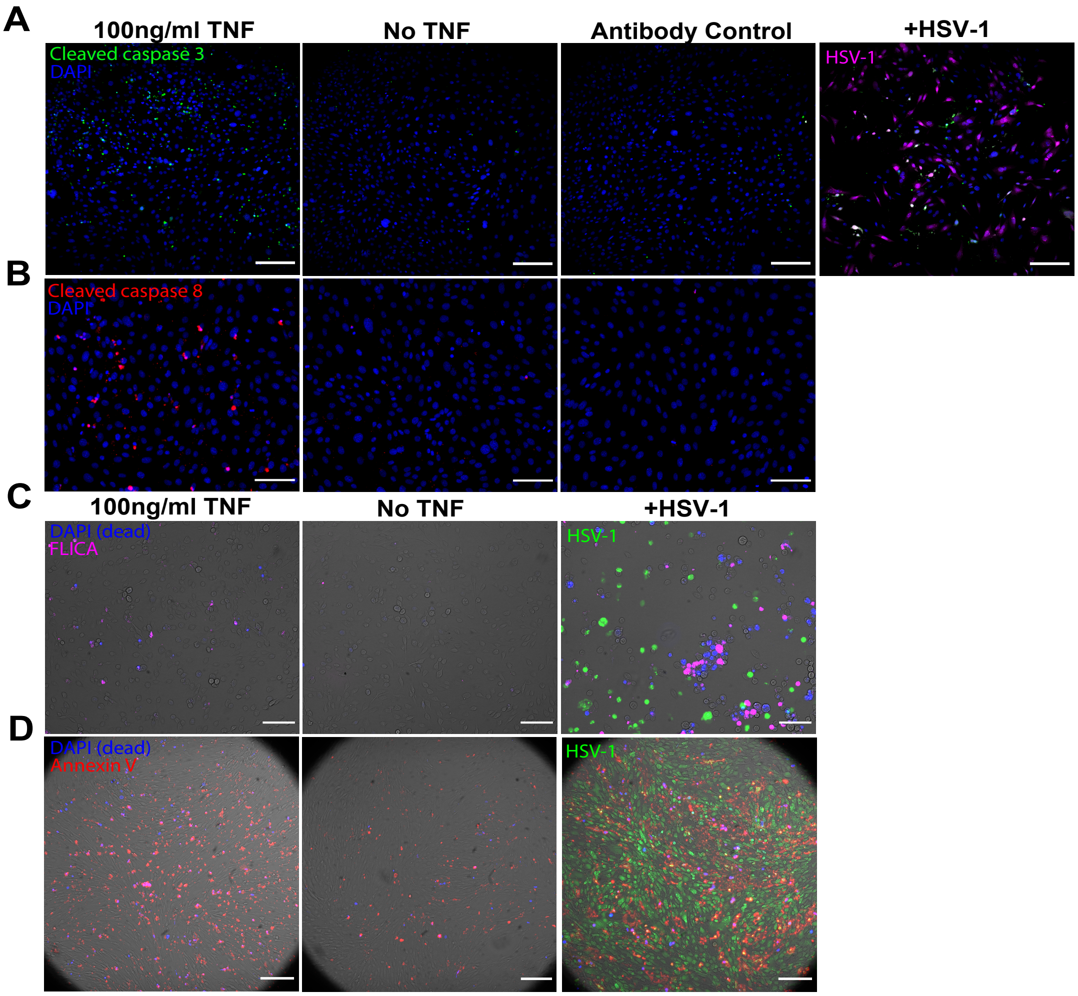

Supplementary Figure 2 (Related to Figure 2): TNF transitions cells into a primed to death cell state. A, Representative images of 3T3 cells stained for cleaved Caspase 3 24h after TNF treatment or infection with MOI 6 of HSV-1. B, Representative images of 3T3 cells stained for cleaved Caspase 8 24h after TNF treatment. C, Representative images of 3T3 cells assayed for caspase activity by FLICA staining 24h after TNF treatment or infection with MOI 10 HSV-1. D, Representative images of 3T3 cells stained for externalized phosphatidylserine by Annexin V staining 24h after TNF treatment or infection with MOI 6 of HSV-1. All scale bars,100µm.

Supplementary Figure 3 (Related to Figure 3): TNF causes the emergence of a speed versus accuracy tradeoff in the cellular decision to die. A, Time courses of cleavage for Caspase 8 activity biosensor. Lines are piecewise linear-fits over a time window of ± 2 frames around each timepoint. 3T3 fibroblasts stably integrated with a Caspase 8 FRET biosensor 21 were treated with TNF and imaged over time. Caspase 8 activity was determined based on changes in the ratio of mVenus to CFP caused by Caspase 8-mediated cleavage to the linker protein. B, Caspase-8 activity measured as the slopes of the lines in panel A. Marker color indicates viability of 3T3 cells as measured using incorporation of DAPI. C, Quantification of the time between the onset of Caspase 8 activity and death. Onset of Caspase 8 activity was determined by applying a 1d step filter and selecting the time of maximal response. D, Distributions of death decision times obtained by single cell tracking of 3T3 cells treated with different doses of TNF and infected with MOI 10 of HSV-1. Orange lines shows an exponential fit of the data. E, Quantification of survival over time for 3T3 cells treated with dose titrations of TNF and infected with MOI 10 HSV-1 or F, left uninfected. Points are data and solid lines represent exponential fits. G, Comparison of death rates quantified from exponential fits of population decay data versus single cell tracking. Points are data and solid line represents a linear fit. H, 3T3 cells were treated with dose titrations of TNF supplemented with either vehicle or 20µM Cisplatin. Cell death was quantified at 48h post treatment. I, 3T3 cells were treated with dose titrations of TNF supplemented with either vehicle or 10µg/ml LPS. Cell death was quantified at 24h post treatment. J, TNF and HSV-1 have a synergistic effect on cell death. We added the death rate for virus infected 3T3 cells and no TNF to the death rates for 3T3 cells treated with TNF alone to estimate additivity (dotted line). The solid line demonstrates the death rates for virus-infected cells treated with TNF is much greater than the expected additivity plot. K, Representative images of 3T3 cells stained for proliferation using Ki-67. L, Quantification of cell proliferation using Ki-67 staining for cells treated with TNF and infected with MOI 10 HSV-1. J,L, Data are mean ± s.d. All scale bars,100µm.

Figure S3

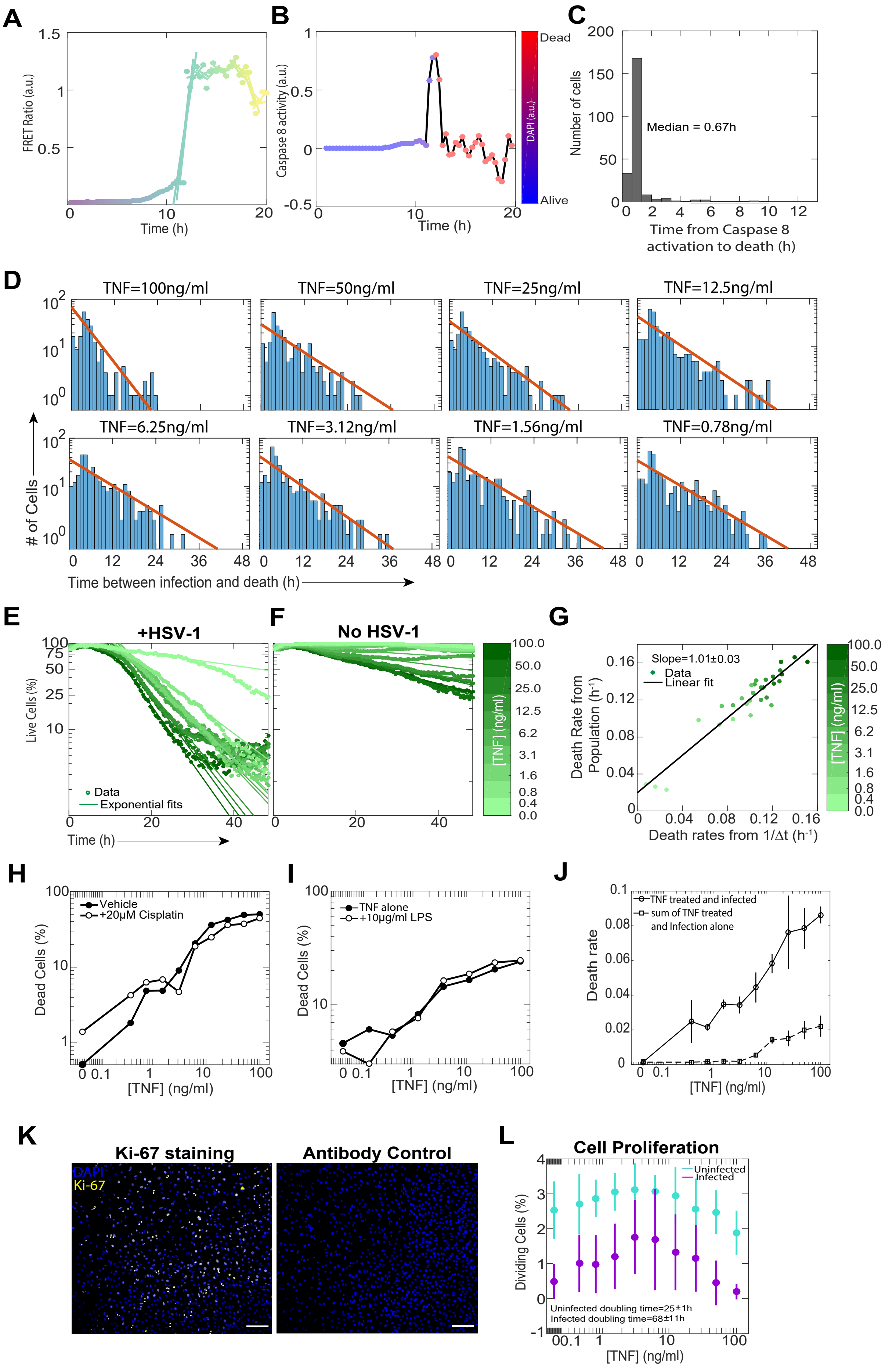
A
FRET Ratio (a.u.)
Time (h)
B
Caspase 8 activity (a.u.)
Time (h)
Dead
Alive
DAPI (a.u.)
C
Number of cells
Median = 0.67h
Time from Caspase 8 activation to death (h)
D
TNF=100ng/ml
TNF=50ng/ml
TNF=25ng/ml
TNF=12.5ng/ml
TNF=6.25ng/ml
TNF=3.12ng/ml
TNF=1.56ng/ml
TNF=0.78ng/ml
# of Cells
Time between infection and death (h)
E
+HSV-1
Live Cells (%)
Data
Exponential fits
Time (h)
F
No HSV-1
[TNF] (ng/ml)
G
Slope=1.01±0.03
Data
Linear fit
Death Rate from Population (h-1)
Death rates from 1/Δt (h-1)
[TNF] (ng/ml)
H
Vehicle
+20μM Cisplatin
Dead Cells (%)
[TNF] (ng/ml)
I
TNF alone
+10μg/ml LPS
Dead Cells (%)
[TNF] (ng/ml)
J
TNF treated and infected
sum of TNF treated and Infection alone
Death rate
[TNF] (ng/ml)
K
Ki-67 staining
Antibody Control
DAPI
Ki-67
L
Cell Proliferation
Uninfected
Infected
Dividing Cells (%)
Uninfected doubling time=25±1h
Infected doubling time=68±11h
[TNF] (ng/ml)

Figure S4

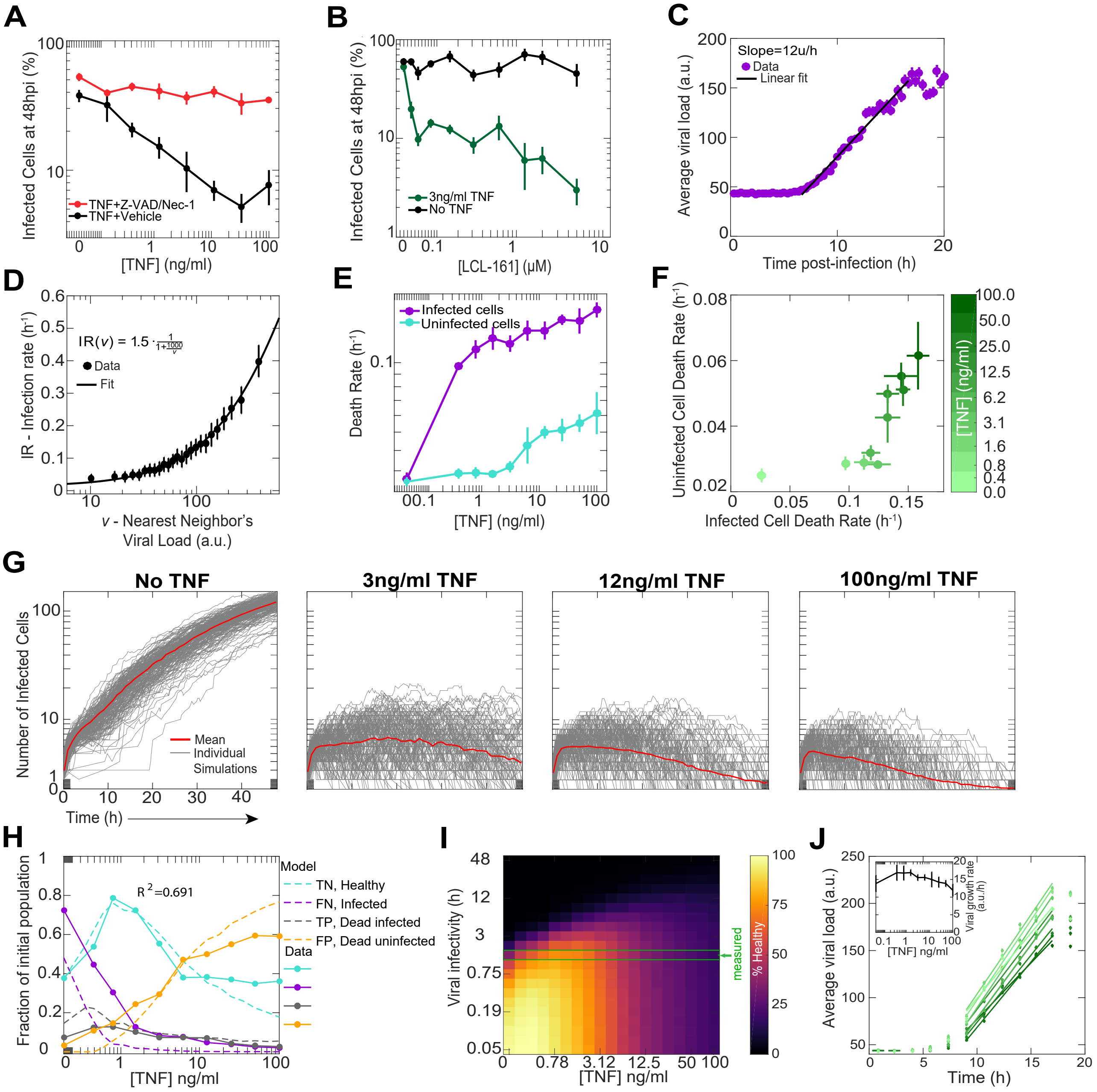

Supplementary Figure 4 (Related to Figure 4): Experimental parameterization of mathematical model. A, Quantification of infected 3T3 cells after treatment with dose titrations of TNF supplemented with vehicle or 30μM Z-Vad and 30μM Necrostatin-1. Cells were infected with MOI 1 of HSV-1. B, Quantification of infected 3T3 cells after treatment with 0 or 3ng/ml TNF supplemented with dose titrations of LCL-161. Cells were infected with MOI 1 of HSV-1. C, Average viral load per cell quantified from HSV-1 fluorescence after infection of 3T3 cells with MOI 10 HSV-1, linear fit of virus accumulation. n≈400 cells. D, Quantification of the infection rate for 3T3 cells based on the viral load of their nearest neighbor. Virus infected cells were cocultured with uninfected targets. Infection in the target cells was measured over time along with the viral load of its neighbors. Infection rate was measured as the probability of a cell to be infected per hour conditioned on a given nearest neighbor viral load. The resulting curve was fit to a Hill function. n≈500 cells. E, Death rates for 3T3 cells treated with dose titrations of TNF and infected with MOI 10 of HSV-1 or left uninfected. F, Quantification of the coupling between infected and uninfected 3T3 cell death rates. G, Simulations of the dynamics of actively infected cells for different doses of TNF. Each condition was simulated 500 times (grey lines). H, Quantification of the percentages of healthy, infected, dead following infection, and dead but uninfected cells treated with dose titrations of TNF and infected with MOI 1 of HSV-1 overlayed with model predictions (R^2=0.69). I. Quantification of the percentages of healthy cells at the end of the simulation for different levels of TNF and viral infectivity. Each condition was simulated 200 times. J. Average viral load per cell quantified from HSV-1 fluorescence after infection of 3T3 cells with MOI 10 HSV-1 in the presence of different doses of TNF, lines denote linear fits of virus accumulation in n≈400 cells. A-B, E-F Data are mean ± s.d. C,D,J Data are mean ± s.e.m.

Figure S5

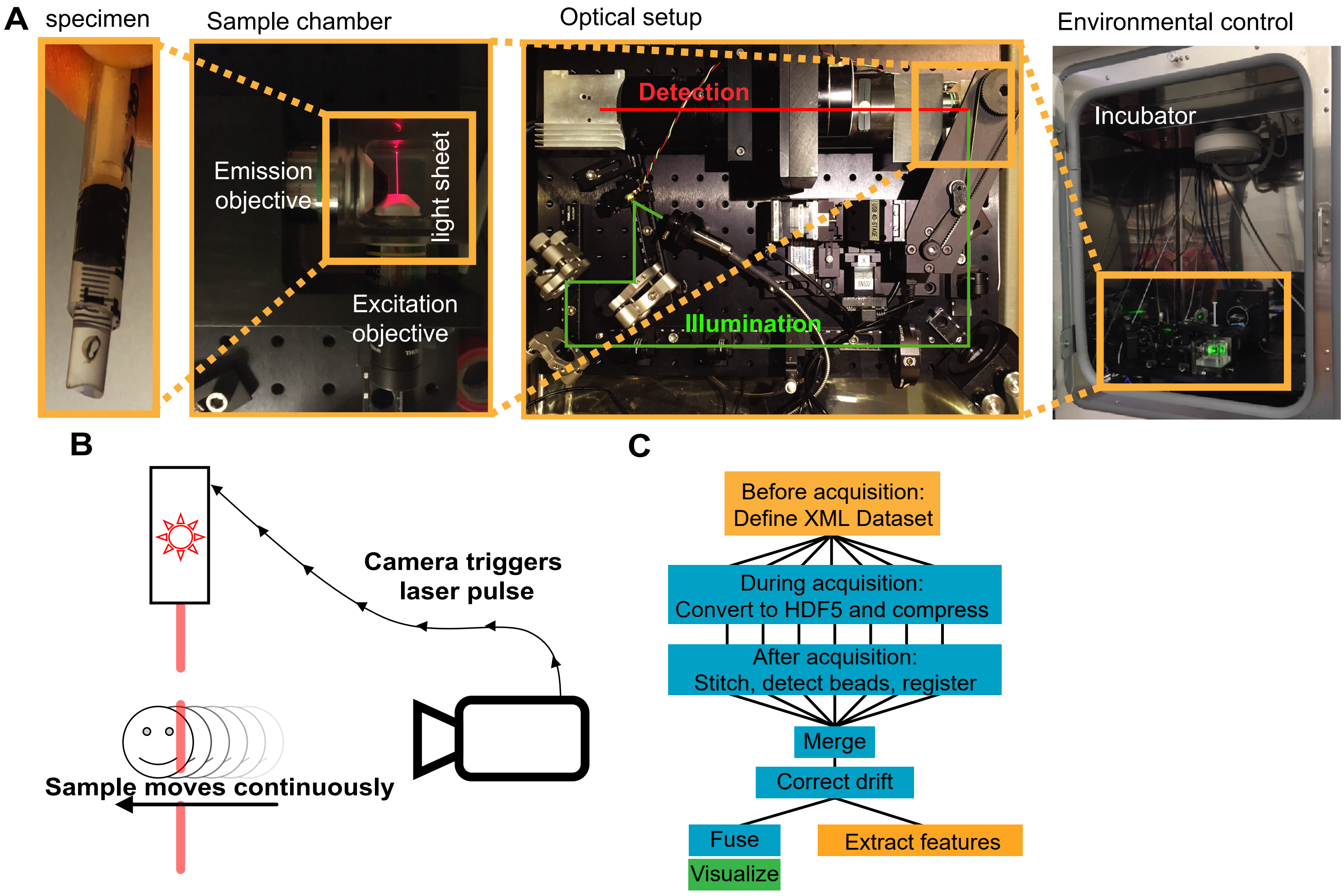

Supplementary Figure 5 (Related to Figure 5): Custom light sheet microscopy setup. A, Specimens are embedded in a 0.5ml syringe in a mixture of 1% low melting point agarose and cornea media. The sample is mounted on a 4D translation-rotation stage and submerged in a sample chamber for imaging. The original optical setup on the OpenSPIM was modified by adding a resonant mirror that provides beam pivoting for even illumination, an optical filter wheel to allow multichannel acquisition, and an alternative emission objective. The whole setup is mounted inside a tissue culture incubator for environmental control. B, To allow fast acquisition, the sample is translated continuously through the light sheet while the camera is acquiring images. At each frame, the camera triggers a short laser pulse. The mechanical stage was upgraded to allow finer translation along the axis of motion. C, Image acquisition and analysis pipeline. Orange boxes implemented in MATLAB, blue boxes use ImageJ and the BigStitcher plugin and implemented as automated bash scripts, and green box implemented using the 3DScript ImageJ plugin.

	value	ci	Rsq		Source
VI(1/h)	1.53	[1.0117 2.0566]	0.9932	viral infectivity rate parameter	S4.D
infection EC50 (a.u.)	1181.2	[666.3 1696.1]	0.9932		S4.C-D
VGR (a.u.)	11.52	[10.9767 12.0742]	0.9777		S4.C-D
VGR (EC50)	0.0108	[0.0048 0.0168]		viral growth rate in units of ec50	S4.C-D
k_p(infected) (1/h)	0.01	[0 0.02]		proliferation rate of infected cells	S3.J
k_p(uninfected)(1/h)	0.027	[0.023, 0.31]		proliferation rate of iuninfected cells	S3.J

TNF	Net Uninfected death rate	Net Infected death rate
0.00	0.0000	0.0159
0.28	0.0000	0.0483
0.39	0.0000	0.0617
0.55	0.0005	0.0744
0.78	0.0014	0.0925
1.10	0.0024	0.0989
1.56	0.0037	0.1079
2.21	0.0055	0.1103
3.13	0.0080	0.1135
4.42	0.0105	0.1161
6.25	0.0140	0.1198
8.84	0.0165	0.1220
12.50	0.0200	0.1250
17.68	0.0220	0.1280
25.00	0.0248	0.1322
35.36	0.0267	0.1351
50.00	0.0293	0.1393
70.71	0.0314	0.1430
100.00	0.0343	0.1481

Death rate - k_p for different TNF concer S4.E, S3.J

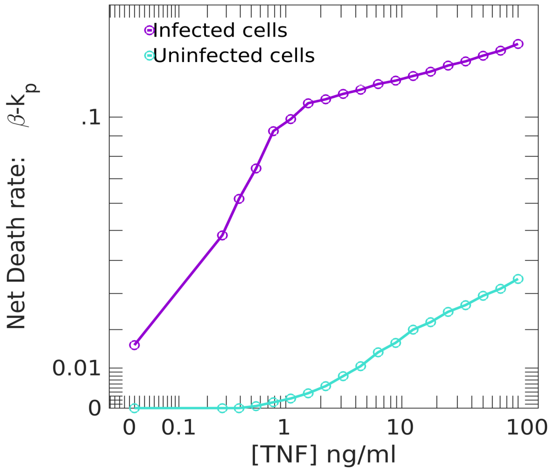

Supplementary Table 1 Model Parameters confidence intervals, and source.

```
function [xOut, loadWhenInf, Rt] =
 SSA_forSpatialSIDGrids_SimpleAnnotated(x,neighMat,...
    VGR,VI,basalDeathRate,infDeathRate)
% inputs are:
%   x - nSpecies * nCells boolean vector i.e.
%       [1 0 0 0 , 0 1 0 0, 1 0 0 0,...]  corresponds to [alive,
 infected, alive, ...]
%   neighMat - nReactions X nReactions matrix specifying which cells
 are nearest neighbors
%   VGR - Viral growth rate in units of infection EC50(S4C-D)
%   VI - Viral infectivity (S4C-D)
%   basalDeathRate - net uninfected death rate for a specific TNF
 concentration
%   infDeathRate - net infected death rate for a specific TNF
 concentration


% Define propensity functions in terms of parameters, k, and states,
 x.
% each cell can be in 4 states - SIDF
w_k_x = @(x,k) [k(1)*x(1), k(2)*x(1), k(3)*x(2)];
% Specify stoichiometry for a single cell
S = [ -1 -1  0 ;...
    1  0 -1 ;...
    0  1  0 ;
    0  0  1];
%reactions: infection, false positive death, infected death

global nReactions nSpecies
nReactions = 3;
nSpecies = 4;

%number of cells
nC = numel(x)/nSpecies;

%make full stoichiometry matrix, block diagonal of single cell stoch
 mat
SCell = repmat({S}, 1, nC);
S = sparse(blkdiag(SCell{:}));

tstop = 48;%finaltime = 48 hours


[xOut, loadWhenInf, Rt] = Run_SSA(w_k_x,S,x,tstop,nC,
 neighMat,VGR,VI,basalDeathRate, infDeathRate); % call code to run
 stochastic simulation.

function [x, loadWhenInf, Rt] =
 Run_SSA(prop_fun,S,x0,tstop,nC,neighMat,VGR,VI,basalDeathRate,
 infDeathRate)

global nReactions nSpecies
```

```
t=0;
x = x0;     %% Specify initial conditions


%Init params
k=zeros(1,nC*nReactions);
k(2:nReactions:end)=basalDeathRate;%basal death rate
k(3:nReactions:end) = infDeathRate; %virus induced death

loadWhenInf = cell(size(neighMat,1),1);
%vector of virus infection. 0 for healthy or dead cells. initial
 infection=1 for infected cells.
v=x(2:nSpecies:end);


while t<tstop
    %update k
    NNvLoad = neighMat*(v(:).*x(2:nSpecies:end)); %nearest neighbor
 viral load in units of EC50
    k(1:nReactions:end) = VI./(1+1./NNvLoad);  %infection probability
 follows hill function (S4.D)

    %Propensity functions: all the probabilities of all the reactions
    w = reshape(cell2mat(arrayfun(@(y,z)
 prop_fun(y{:},z{:}), mat2cell(x(:),nSpecies*ones(1,nC)),
 mat2cell(k(:),nReactions*ones(1,nC)),'uniformoutput', false))',1,
[]);%sorry for the oneliner
    w0 = sum(w);                              % sum of the prop.
 functions
    dt = 1/w0*log(1/rand);           %when's the next reaction?
    t = t+dt;                        % update time of next reaction,
 exp dist
    if t<=tstop
        r2w0=rand*w0;                % generate second random number
 and multiply by prop. sum
        i=1;                                            % initialize
 reaction counter
        while sum(w(1:i))<r2w0             % what's the next reaction?
 increment counter until sum(w(1:i)) exceeds r2w0
            i=i+1;
        end
        x = x+S(:,i);                                   % update the
 configuration (apply reaction)

        if mod(i,3)==1 %for Rt, if a cell gets infected, keep it's nn
 viral load
            loadWhenInf{ceil(i/3)} = neighMat(ceil(i/3),:).*v';
        end
        v = v+VGR*dt*x(2:nSpecies:end);                        %
 increase viral load for infected live cells
    end

end
```

```
NNLoad = loadWhenInf;
C1 = cellfun(@(x) x./sum(x), NNLoad, 'UniformOutput', false);
indsOfCellsThatGotInfected = find(~cellfun(@isempty, NNLoad));

cellsIInfected = zeros(size(NNLoad));

for i=indsOfCellsThatGotInfected'
    [~,ind,prob] = find(C1{i});
    cellsIInfected(ind) = cellsIInfected(ind) + prob';
end

a = k(1:nReactions:end)./ infDeathRate; %probability of getting
 infected devided by probability of infecting cell to die gives Rt
 estimate
a(a==0)=[];
Rt = mean(a);
if isnan(Rt)
    R0=0;
end
```

Published with MATLAB® R2016b
