# Supplementary material for: TNF controls a speed-accuracy tradeoff in the cell death decision to restrict viral spread
Source: Nat Commun. 2021 May 20;12:2992. doi: 10.1038/s41467-021-23195-9 (PMC8137918; doi:10.1038/s41467-021-23195-9)
Supplement: Supplementary file 3 — Descriptions of Additional Supplementary Files [file 41467_2021_23195_MOESM3_ESM.pdf]

**Descriptions of Additional Supplementary Files**

**Supplementary Movie 1**

**Description:** HSV-1 spread with Activated macrophages**.** 3T3 fibroblasts were co-cultured with 50% activated, inflammatory macrophages and infected with MOI 1 of HSV-1. Cells were imaged continuously for around 3 days. Left panel: Viral spread, HSV-1 shown in purple and cell nuclei in cyan. Right panel: Viral spread from left panel color-coded based on time of infection. Scale bar, 100μm.

**Supplementary Movie 2**

Description: HSV-1 spread with Naive macrophages. 3T3 fibroblasts were co-cultured with 50% naive macrophages and infected with MOI 1 of HSV-1. Cells were imaged continuously for around 3 days. Left panel: Viral spread, HSV-1 shown in purple and cell nuclei in cyan. Right panel: Viral spread from left panel color-coded based on time of infection. Scale bar, 100μm.

**Supplementary Movie 3**

**Description:** HSV-1 spread with Activated TNF KO macrophages. 3T3 fibroblasts were co-cultured with 50% activated, inflammatory TNF KO macrophages and infected with MOI 1 of HSV-1. Cells were imaged continuously for around 3 days. Left panel: Viral spread, HSV-1 shown in purple and cell nuclei in cyan. Right panel: Viral spread from left panel color-coded based on time of infection. Scale bar, 100μm.

**Supplementary Movie 4**

**Description:** HSV-1 spread in the absence of macrophages. 3T3 fibroblasts were infected with MOI 1 of HSV-1. Cells were imaged continuously for around 3 days. Left panel: Viral spread, HSV-1 shown in purple and cell nuclei in cyan. Right panel: Viral spread from left panel color-coded based on time of infection. Scale bar, 100μm.

**Supplementary Movie 5**

**Description:** Light-sheet imaging of HSV-1 spread in whole, live mouse corneas. R26-H2B-mCherry mouse corneas were infected with HSV-1 embedded in agarose and imaged for 48 hours. HSV-1 is shown in purple and dead cells are shown in orange. Scale bar, 100μm.

**Supplementary Movie 6**

**Description:** Light-sheet imaging of HSV-1 spread in whole, live TNF-treated mouse corneas. R26-H2B-mCherry mouse corneas were infected with HSV-1, embedded in agarose with 50ng/ml TNFα and imaged for 48 hours. HSV-1 is shown in purple and dead cells are shown in orange. Scale bar, 100μm.
