# Supplementary material for: TNF controls a speed-accuracy tradeoff in the cell death decision to restrict viral spread
Source: Nat Commun. 2021 May 20;12:2992. doi: 10.1038/s41467-021-23195-9 (PMC8137918; doi:10.1038/s41467-021-23195-9)
Supplement: Supplementary file 10 — Reporting Summary [file 41467_2021_23195_MOESM10_ESM.pdf]

nature research

Corresponding author(s): Roy Wollman

Last updated by author(s): March 17, 2021

Reporting Summary

Nature Research wishes to improve the reproducibility of the work that we publish. This form provides structure for consistency and transparency in reporting. For further information on Nature Research policies, see our Editorial Policies and the Editorial Policy Checklist.

Statistics

For all statistical analyses, confirm that the following items are present in the figure legend, table legend, main text, or Methods section.

n/a	Confirmed	
☐	☒	The exact sample size (*n*) for each experimental group/condition, given as a discrete number and unit of measurement
☐	☒	A statement on whether measurements were taken from distinct samples or whether the same sample was measured repeatedly
☒	☐	The statistical test(s) used AND whether they are one- or two-sided<br>*Only common tests should be described solely by name; describe more complex techniques in the Methods section.*
☒	☐	A description of all covariates tested
☐	☒	A description of any assumptions or corrections, such as tests of normality and adjustment for multiple comparisons
☐	☒	A full description of the statistical parameters including central tendency (e.g. means) or other basic estimates (e.g. regression coefficient) AND variation (e.g. standard deviation) or associated estimates of uncertainty (e.g. confidence intervals)
☒	☐	For null hypothesis testing, the test statistic (e.g. *F*, *t*, *r*) with confidence intervals, effect sizes, degrees of freedom and *P* value noted<br>*Give P values as exact values whenever suitable.*
☒	☐	For Bayesian analysis, information on the choice of priors and Markov chain Monte Carlo settings
☒	☐	For hierarchical and complex designs, identification of the appropriate level for tests and full reporting of outcomes
X	☐	Estimates of effect sizes (e.g. Cohen's *d*, Pearson's *r*), indicating how they were calculated

Our web collection on statistics for biologists contains articles on many of the points above.

Software and code

Policy information about availability of computer code

Data collection	All microscopy data was acquired using Micro-Manager (2.0) and custom code written in MATLAB (2020a) and is available at https://github.com/wollmanlab/Scope, https://github.com/wollmanlab/Metadata , https://github.com/wollmanlab/bigstitchparallel
Data analysis	All data analysis was done using custom code written in MATLAB ans is available at https://github.com/wollmanlab/SingleCellVirusProcessing , https://github.com/wollmanlab/Results

For manuscripts utilizing custom algorithms or software that are central to the research but not yet described in published literature, software must be made available to editors and reviewers. We strongly encourage code deposition in a community repository (e.g. GitHub). See the Nature Research guidelines for submitting code & software for further information.

Data

Policy information about availability of data

All manuscripts must include a data availability statement. This statement should provide the following information, where applicable:

- Accession codes, unique identifiers, or web links for publicly available datasets
- A list of figures that have associated raw data
- A description of any restrictions on data availability

Source data that support the findings of this study are provided with this paper and original images can be obtained from the corresponding author upon reasonable request.

Field-specific reporting

Please select the one below that is the best fit for your research. If you are not sure, read the appropriate sections before making your selection.

☒ Life sciences ☐ Behavioural & social sciences ☐ Ecological, evolutionary & environmental sciences

For a reference copy of the document with all sections, see nature.com/documents/nr-reporting-summary-flat.pdf

Life sciences study design

All studies must disclose on these points even when the disclosure is negative.

Sample size	Each experiment includes approximately 500 cells per field of view, and between 2-4 technical replicates per experiment, leading to quantifications based on 1000-2000 cells per experiment. Each experiment was repeated a minimum of three independent times. This results in a robust number of cells from which to draw conclusions between treatment types and conditions. Sample size (typically in the thousands) were chosen based on microscope throughput to be large enough to avoid concerns related to sample size.
Data exclusions	We did not exclude data from this study.
Replication	All experiments were performed a minimum of three separate times (and often more than 3 times). No experiments were discarded. There is a statement confirming this in the text.
Randomization	Cells and mice were randomly allocated into experimental groups.
Blinding	Blinding was not relevant to this study as the impact as no treatment was tested.

Reporting for specific materials, systems and methods

We require information from authors about some types of materials, experimental systems and methods used in many studies. Here, indicate whether each material, system or method listed is relevant to your study. If you are not sure if a list item applies to your research, read the appropriate section before selecting a response.

Materials & experimental systems

n/a	Involved in the study	
☐	☒	Antibodies
☐	☒	Eukaryotic cell lines
☒	☐	Palaeontology and archaeology
☐	☒	Animals and other organisms
☒	☐	Human research participants
☒	☐	Clinical data
☒	☐	Dual use research of concern

Methods

n/a	Involved in the study	
☒	☐	ChIP-seq
☒	☐	Flow cytometry
☒	☐	MRI-based neuroimaging

Antibodies

Antibodies used	Monoclonal anti-Cleaved Caspase 8 (clone D5B2) (Cell Signaling Technologies), monoclonal anti-Cleaved Caspase 3 (clone D175) (Cell Signaling Technologies), monoclonal anti-phospho-STING (clone D1C4T) (Cell Signaling Technologies), monoclonal anti-TNFα (clone MP6-XT22) (Biolegend), and monoclonal anti-Ki-67 (clone 16A8) (Biolegend). The secondary antibodies used were (all from Jackson ImmunoResearch): polyclonal goat anti-Rabbit IgG-Alexa 594 (cat: 111-586-003), polyclonal goat anti-Rabbit IgG-Alexa 647 (cat: 111-606-003), and polyclonal goat anti-Rat IgG-Alexa 488 (cat: 112-546-003).<br><br>To detect externalized phosphatidylserine by Annexin V and Caspase 8 activity by FAM-FLICA-FMK, we used Annexin V-Alexa 568 (Thermo Fisher) and the Vybrant FAM Caspase 8 (Thermo Fisher) Assay kit according to manufacturer instructors.
Validation	Validation of antibodies was done by including negative and positive controls for all antibodies used. Images and detailed explanation of validation procedure, the controls and their descriptions are shown in the supplementary figures.

Eukaryotic cell lines

Policy information about cell lines

Cell line source(s)	ATCC
Authentication	Cell lines were authenticated by ATCC. We did not authenticate the cells lines otherwise

Mycoplasma contamination	Mycoplasma testing is performed in the lab on a twice-annual basis. We did NOT find any mycoplasma
Commonly misidentified lines (See ICLAC register)	We do not make use of any commonly misidentified cell lines as described in the ICLAC register.

Animals and other organisms

Policy information about studies involving animals; ARRIVE guidelines recommended for reporting animal research

Laboratory animals	TNF+/+-RelA-Venus and TNF-/--RelA-Venus mice (C57Bl/6J background); C57Bl/6J; Rosa26-H2B-mCherry-/+ (C57Bl/6 background). Both male and female mice were used and all were within the age range of 8-15 weeks. Animals were housed according to approved IACUC protocols.
Wild animals	This study did not include wild animals.
Field-collected samples	This study did not involve samples collected from the field.
Ethics oversight	All animal experiments were approved by the UCLA Animal Research Committee and mice were maintained in group housing at an AALAC accredited animal facility in standard SPF conditions.

Note that full information on the approval of the study protocol must also be provided in the manuscript.
